# Supplementary material for: Comparative Genomics of DH5α-Inhibiting Escherichia coli Isolates from Feces of Healthy Individuals Reveals Common Co-Occurrence of Bacteriocin Genes with Virulence Factors and Antibiotic Resistance Genes
Source: Antibiotics (Basel). 2025 Aug 26;14(9):860. doi: 10.3390/antibiotics14090860 (PMC12466380; doi:10.3390/antibiotics14090860)
Supplement: Supplementary file 1 [file antibiotics-14-00860-s001.zip › antibiotics-3791638-supplementary.pdf]

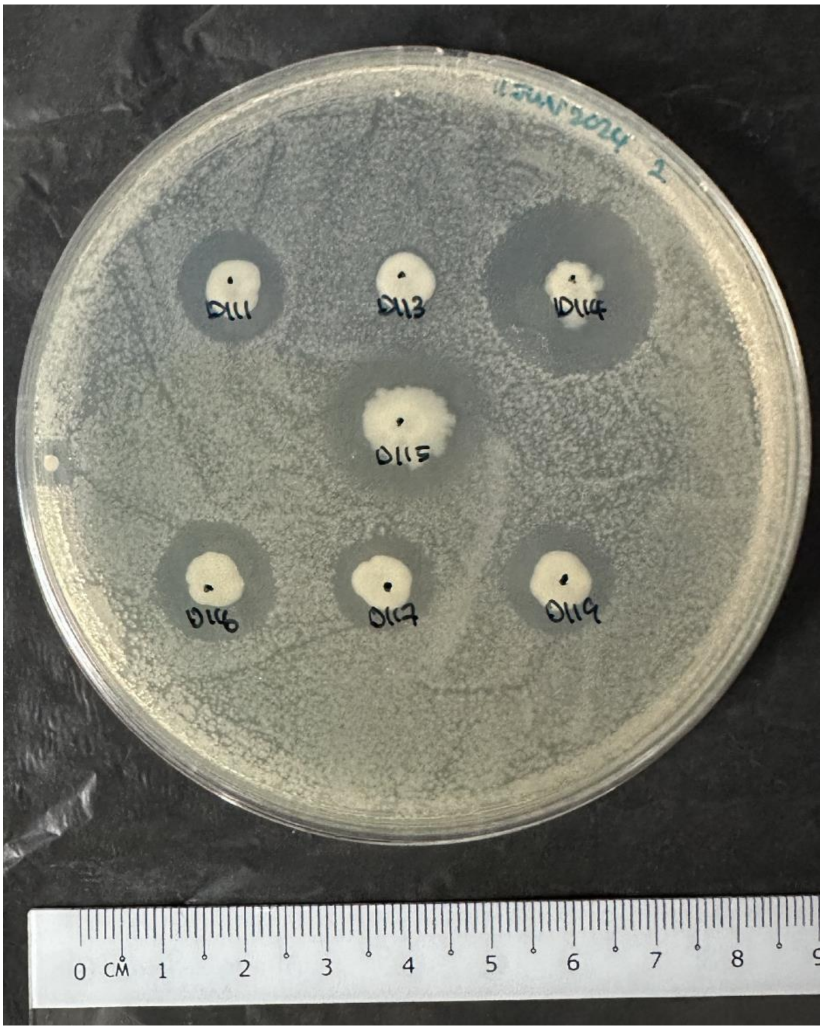

Supporting Figure 1. Representative image of spot-on-lawn assay. Colonies of inhibitory strains were picked and spotted onto a lawn of *Escherichia coli* DH5*α*, which was chosen as it is non-pathogenic and also non-bacteriocin producing.
